# Oncological Efficacy of Robotic Nephroureterectomy vs. Open and Laparoscopic Nephroureterectomy for Suspected Non-Metastatic UTUC—A Systematic Review and Meta-Analysis

**DOI:** 10.3390/cancers15204926

**Published:** 2023-10-10

**Authors:** Karthik Rajan, Ahmad Khalifa, Robert Geraghty, Kalpesh Parmar, Gokul KandaSwamy, Juan Gómez Rivas, Bhaskar Somani, Bhavan Prasad Rai

**Affiliations:** 1 Department of Urology, Freeman Hospital, Newcastle NE7 7PJ, UK; 2 Department of Urology, Morriston Hospital, Swansea SA6 6NL, UK; 3 Department of Urology, Hospital Clínico San Carlos, 28040 Madrid, Spain; 4 Department of Urology, University Hospital Southampton, Southampton SO16 6YD, UK

**Keywords:** upper tract urothelial carcinoma, radical nephroureterectomy, robotic nephroureterectomy, laparoscopic nephroureterectomy, open nephroureterectomy, neoplasms of ureter, robotic surgery, minimally invasive techniques

## Abstract

**Simple Summary:**

Upper tract urothelial cancer is an aggressive malignancy that requires prompt treatment in the form of removal of the kidney and ureter with a bladder cuff, especially for invasive disease. The ideal surgical technique should offer a complete cancer clearance, including the ability to remove lymph glands with a short recovery period. The three techniques currently available include open, laparoscopic, and robotic approaches. This review compares the three techniques both on cancer clearance and survival by analyzing the existing literature. We found the robotic technique to be similar or slightly superior to either the open or laparoscopic approach for overall survival, cancer-specific survival, lymph node removal rates, and the rates of risk for residual cancer (positive margins). However, the risk of recurrent tumours in the bladder was identified to be higher in robotic surgery compared to the open approach. The study determines that the robotic approach offers comparable outcomes to the open and laparoscopic approaches, which are well established but for which the overall quality of evidence has been poor.

**Abstract:**

Introduction and Aims: The optimal approach for nephroureterectomy in patients with suspected UTUC remains a point of debate. In this review, we compare the oncological outcomes of robotic nephroureterectomy (RNU) with open (ONU) or laparoscopic nephroureterectomy (LNU). Methods: All randomized trials and observational studies comparing RNU with ONU and/or LNU for suspected non-metastatic UTUC are included in this review. The systematic review was performed in accordance with the Cochrane Guidelines and the Preferred Reporting Items for Systematic Reviews and Meta-Analyses (PRISMA). The primary outcome measures were overall survival (OS), cancer-specific survival (CSS), disease-free survival (DFS), and intravesical recurrence-free survival (IV-RFS). The secondary outcome measures were the lymph node dissection (LND) rates, positive margin rates, and the proportion of patients receiving bladder intravesical chemotherapy. Results: We identified 8172 references through our electronic searches and 8 studies through manual searching. A total of 15 studies met the inclusion criteria. The total number of patients in the review was 18,964. RNU had superior OS compared to LNU (HR: 0.81 (95% CI: 0.71, 0.93), *p*-0.002 (very low certainty)). RNU and ONU had similar OS (HR: 0.83 (95% CI: 0.52, 1.34), *p*-0.44 (very low certainty)). One study reported an independent association of RNU as a worse predictor of IV-RFS when compared to ONU (HR-1.73 (95% CI: 1.22, 2.45)). The LND rates were higher in the RNU cohort when compared to the LNU cohort (RR 1.24 (95% CI: 1.03, 1.51), *p*-0.03 (low certainty)). The positive margin rate was lower in the RNU cohort when compared to the ONU cohort (RR 0.29 (95% CI: 0.08, 0.86), *p*-0.03 (low certainty)). Conclusion: RNU offers comparable oncological efficacy to ONU, except for intravesical recurrence-free survival (IV-RFS). RNU has fewer positive surgical margin rates compared to ONU in well-balanced studies. RNU appears to outperform LNU for certain oncological parameters, such as OS and the proportion of patients who receive lymph node dissections. The quality of evidence comparing surgical techniques for UTUC has remained poor in the last decade.

## 1. Introduction

Upper tract urothelial cancers (UTUCs) are characterized by their aggressive nature with relatively modest survival outcomes, most commonly necessitating a radical nephroureterectomy (NU) with or without a lymphadenectomy (LND), particularly for invasive or high-grade disease [1]. The optimal surgical approach for performing a nephroureterectomy (NU) has been the subject of an ongoing debate [2]. Crucial oncological considerations with surgical approaches to NU include sustaining oncological efficacy, reducing metachronous bladder cancer recurrence rates, ensuring a robust bladder cuff excision (BCE), the feasibility of lymph node dissection (LND), and optimizing the administration of intravesical and systemic anti-cancer therapies [1]. It is, therefore, vital to devise management strategies for patients who are not only oncologically efficacious but also have acceptable toxicity, ensuring a wider cohort of beneficiaries. Previous reports have suggested that open nephroureterectomy (ONU) exhibits superior oncological efficacy compared to laparoscopic nephroureterectomy (LNU) in patients with locally advanced disease [1]. Simone et al. [3], in a prospective randomized control trial, reported that the cancer-specific survival and metastasis-free survival of patients who underwent ONU was significantly better than LNU when matched for T3 or high-grade disease. Potential reasons for these observations include sub-optimal techniques of dealing with the bladder cuff and technical challenges with LND during an LNU [4]. ONU is, however, a morbid procedure, limiting the proportion of patients who will tolerate such an intervention [5]. It is, therefore, vital that we refine minimally invasive techniques that can achieve comparable oncological efficacy to ONU [5]. Robotic technology has the potential to address some of the aforementioned limitations associated with the conventional laparoscopic technique of NU. The purported benefits of the robotic technology have led to a notable global adoption of robotic nephroureterectomy (RNU) over the past decade in anticipation of enhanced oncological outcomes. This systematic review aims to meticulously evaluate the contemporary literature, comparing the robotic technique with the laparoscopic and open NU techniques for suspected UTUC, with an emphasis on the oncological outcomes.

## 2. Methods

### 2.1. Evidence Acquisition

#### 2.1.1. Criteria for Considered Studies in This Review

*Types of Studies:* All randomized trials and observational studies comparing robotic nephroureterectomy (RNU) with open (ONU) and/or laparoscopic nephroureterectomy (LNU).

*Types of participants:* Adult participants with a suspected non-metastatic UTUC.

#### 2.1.2. Search Strategy and Study Selection

The review protocol was registered with PROSPERO 2023 CRD42023418801. The systematic review was performed in accordance with the Cochrane Guidelines and the Preferred Reporting Items for Systematic Reviews and Meta-Analyses (PRISMA) [6,7]. The bibliographic databases searched were Embase, Medline, Cochrane Library, Web of Science, CINAHL, British Nursing Index, LILACS, Biomed Central, BIOSIS, Scopus, and Amed. The search was conducted in May 2023. All studies comparing RNU with ONU and LNU were evaluated. See Supplementary File S1 for the search terms used for the strategies in this review.

### 2.2. Outcomes Measures

#### 2.2.1. Primary—Time-to-Event Analysis

- Overall survival (OS);
- Cancer-specific survival (CSS);
- Disease-free survival (DFS);
- Intravesical recurrence-free survival (IV-RFS).

#### 2.2.2. Secondary

- Lymph node dissection rates;
- Positive margin rates;
- Proportion of patients receiving bladder intra-vesical chemotherapy.

### 2.3. Quality Assessment of Evidence and Certainty of Outcomes

The risk of bias was assessed by using the recommended tool in the Cochrane Handbook for Systematic Reviews of Interventions under the following domains: random sequence generation, allocation concealment, blinding of participants and personnel, blinding of outcome assessment, incomplete outcome data, and selective reporting [8]. Due to the inherently higher risk of selection bias in non-randomized studies (NRS), the Cochrane risk of bias tool was assessed for pre-specified confounders (general condition: performance status, ASA score, BMI, smoking status, primary grade and clinical stage, location of the tumor, focality, previous ureteroscopy). The Grading of Recommendations Assessment, Development and Evaluation (GRADE) approach was used to rate the quality of evidence for each outcome [9].

### 2.4. Data Extraction and Analysis

Two reviewers (B.P.R. and K.R.) independently identified all studies that appeared to fit the inclusion criteria for full review. Disagreement was resolved by consensus. For NRS, a pooled analysis for continuous and dichotomous data was performed if the cohorts were balanced and judged as having a low risk of selection bias. For the time-to-event analysis of OS, CSS, and DFS, a pooled analysis was performed if a Cox regression multivariate analysis included TNM staging as a covariate. For the time-to-event analysis of IV-RFS, a pooled analysis was performed if a Cox regression multivariate analysis was performed by the primary studies, taking into consideration diagnostic URS and previous bladder cancer history. If the data available did not fulfill the aforementioned criteria, a pooled analysis was not performed. These data were described narratively and presented in forest plots. Where pooled analysis was performed, we used the Mantel–Haenszel method, the inverse variance method, and the generic inverse variance method for the dichotomous, continuous, and time-to-event outcomes, respectively. We imputed the means and standard deviations from the median, range, and interquartile range following the guidance provided in previous studies [10,11,12]. *p*-values were considered significant if <0.05. A random-effects model was used to summarize the pooled analysis. The heterogeneity was analyzed using a Chi-squared test on N-1 degrees of freedom, with an alpha of 0.05 used for statistical significance, and with the I^2^ statistic. I^2^ values of 0% to 40%, 30% to 60%, 50% to 75%, and 90% to 100% were interpreted as “may not be important”, “moderate heterogeneity”, “substantial heterogeneity”, and “considerable heterogeneity”, respectively. Cochrane Review Manager Software Version 5.4 was used to perform the statistical analysis.

## 3. Results

**Figure 1:**
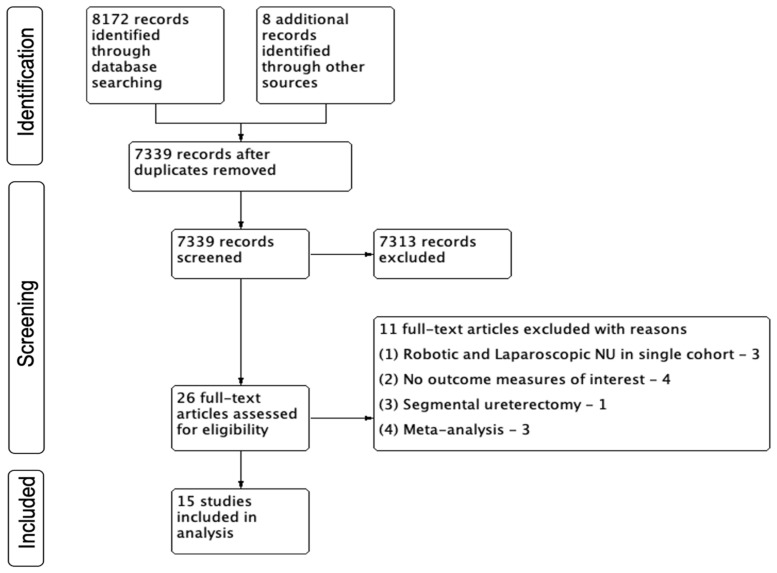
PRISMA Chart.

The study selection process is described using a PRISMA flow diagram in Figure 1. We identified 8172 references through our electronic searches and 8 studies through manual searching. We retrieved a total of 26 references for a comprehensive full-text evaluation.

A total of 15 studies met the inclusion criteria of this review [13,14,15,16,17,18,19,20,21,22,23,24,25,26,27]. The study by Mourmouris et al. [22] was the only one that was reported as a prospective study from two institutions. Additionally, three studies reported outcomes from large multi-institutional consortiums [21,24,26], and three studies reported data from the cancer registry National Cancer Database based in the United States of America (USA) [16,18,20]. A propensity-based matched pair analysis was performed in three studies [23,24,26] (Table 1).

The total number of patients in the review was 18,964. The numbers of patients stratified to the RNU, LNU, and ONU cohorts were 5085, 9143, and 4736 respectively. The demographic data and tumor characteristics of the individual studies are summarized in Table 2 and Table 3, respectively.

### 3.1. Primary Outcome: Time-to-Event Analysis (Table 4)

#### 3.1.1. Overall Survival (OS) (Figure 2)

- RNU vs. LNU

A total of four studies had comparative data considered suitable for a pooled analysis [20,21,25,27]. RNU had superior OS compared to LNU (HR: 0.81 (95% CI: 0.71, 0.93), I^2^ = 0%, *p*-0.002 (very low certainty)).

**Table 4:** Survival outcomes of included studies.

Author	Follow-Up (Months)	Survival Outcomes
Rodriguez et al. 2017 [16]	ND	ONU vs. RNU—HR, CI, *p*-value OS RNU—0.88, (0.71–1.09), *p* = 0.227
Lee et al., 2019 [17] (included in SR)	RNU vs. LNU vs. ONU 23.7 ± 2.1 vs. 38.1 ± 3.3 vs. 41.7 ± 3.3 (*p* < 0.001) (Mean, SD)	Multivariate analysis: ONU vs. RNU (HR (95% CI), *p*-value) Intravesical recurrence-free survival (IV-RFS)—0.665 (0.405–1.092), 0.107; Overall mortality—0.335 (0.097–1.158), 0.084; Cancer-specific mortality—0.336 (0.070–1.607), 0.172; Disease-free survival (DFS)—0.574 (0.276–1.325), 0.326. Multivariate Cox proportional hazard analyses were used to reveal the predictors of postoperative survival outcomes using the following covariates: age, sex, BMI, ECOG score, T stage, tumor size, tumor grade, Charlson’s comorbidity index, multifocality of the tumor, lymphadenectomy, and lymph node invasion.
Ye et al. 2020 [19] Balanced for - Primary grade and clinical stage; - Location of the tumor; - Previous ureteroscopy; - No data on other confounders.	RNU vs. LNU 40.5 vs. 40.4 (Median)	RNU vs. LNU 5-year OS 67.4% vs. 84.0%, (*p* = 0.524); 5-year CSS 71.2% vs. 84.7%, (*p* = 0.728); 5-year IV-RFS 88.0% vs. 85.5%, (*p* = 0.611); 5-year retroperitoneal recurrence-free survival (77.3% vs. 87.7%, *p* = 0.737); 5-year metastasis-free survival (93.1% vs. 96.7%, *p* = 0.323).
Kenigsberg et al. 2021 [20] (included in SR)	RNU vs. LNU 33.3 vs. 35.1 (*p* = 0.063) (Mean)	RNU vs. LNU Kaplan–Meier survival analysis Median OS—71.1 months vs. 62.6 months, *p* = 0.033 Multivariate analysis All-cause mortality LNU worse than RNU → HR- 1.182, 95% CI 1.016–1.375, *p* = 0.030 Multivariate Cox proportional hazard analyses were used to reveal the predictors of postoperative survival outcomes using the following covariates: age, sex, year of diagnosis, Charlson’s comorbidity index, cellular grade, ECOG score, T stage, N stage, positive surgical margin, lymphadenectomy, neoadjuvant chemotherapy.
Li et al. 2021 [21]	ND	Hand-assisted LNU vs. LNU vs. RNU 5-year OS 71% 74% vs. 82% (*p* = 0.010); 5-year CSS 80% vs. 86% vs. 87% (*p* = 0.037). Hand-assisted LNU vs. RNU—multivariate analysis, HR (95%CI), *p*-value OS—0.534 (0.318, 0.896), *p* = 0.018; CSS 0.730 (0.413, 1.290), *p* = 0.279; IV-RFS 1.082 (0.751, 1.558), *p* = 0.673. Multivariate Cox proportional hazard analyses were used to reveal the predictors of postoperative survival outcomes using the following covariates: age, sex, stage of chronic kidney disease, cytology, history of bladder cancer, hydronephrosis, tumor location, tumor grade, tumor size, multifocality, ECOG score, T stage, N stage, histological variant, tumor necrosis.
Zeuschner et al. 2021 [23] Propensity matched analysis—balanced for - pT stage; - Tumor location; - Prior cystectomies.	Overall cohort 30.9 (1.4–129.5) (Median, range)	RNU vs. ONU 5-year OS 59.1% (39.0–74.5) vs. 46.9% (27.9–64.5) *p* = 0.087; 5-year progression-free survival (PFS) 47.9% (27.8–65.4) vs. 38.0% (20.5–55.4) *p* = 0.132. No difference in OS and PFS between RNU and ONU in multivariate analysis. Multivariate Cox proportional hazard analyses were used to reveal the predictors of postoperative survival outcomes using the following covariates: age, sex, BMI, previous cystectomy, simultaneous cystectomy, bladder cuff excision, lymphadenectomy, locally advanced tumor, pR1, pN+.
Bae et al. 2022 [25] (included in SR)	RNU vs. LNU vs. ONU 22 ± 12.4 vs. 29.92 ± 15.3 vs. 32.4 ± 16.4 (*p* < 0.001) (Mean, SD)	RNU vs. LNU vs. ONU 3-year OS—92.1% vs. 90.4% vs. 91.8%, *p* = 0.819; 3-year DFS- 80.9% vs. 74.2% vs. 77.1%, *p* = 0.842. Multivariate Cox proportional hazards of robotic—reference vs. lap, open—HR (95% CI), *p* value PFS—1.45 (0.68–3.11), 1.29 (0.71–2.33), *p* = 0.580; CSS—0.98 (0.22–4.40), 1.12 (0.35–3.54), *p* = 0.970; OS—0.77 (0.18–3.30), 1.28 (0.45–3.61), *p* = 0.699. Multivariate Cox proportional hazard analyses were used to reveal the predictors of postoperative survival outcomes using the following covariates: age, pT staging ≥T3, pN+, positive surgical margin, presence of LVI, tumor grade III, adjuvant chemotherapy.
Grossmann et al. 2023 [26] (included in SR)	Overall cohort 32 (15–61) (Median, IQR)	RNU vs. LNU vs. ONU—(95% CI), *p* value 3-year OS—80.9%(73.8–88.6) vs. 75.1(69.3–81.2) vs. 76.9(71.4–82.7), *p* = 0.7; 3-year CSS—85.5%(79.1–92.5) vs. 84.1%(79.2–89.4) vs. 86.5 (81.9–91.3), *p* = 0.7; 3-year DFS—72.9%(66–80.6) vs. 77.6% (72.2–83.4) vs. 73.5% (67.9–79.7), *p* = 0.7; 3-year IV-RFS—58.9%(51.9–66.7) vs. 58.8(52.5–65.8) vs. 73.5(67.7–79.8)—Improved IV-RFS for open in pairwise log rank *p* < 0.001. Multivariate Cox regression, ONU vs. RNU—HR (95% CI), *p* value RFS → 1.03 (0.71–1.49), *p* = 0.9; IV-RFS → 1.73 (1.22–2.47), *p* = 0.002; CSS → 0.65 (0.39–1.10), *p* = 0.1; OS → 0.81 (0.55–1.19), *p* = 0.3. Multivariate Cox proportional hazard analyses were used to reveal the predictors of postoperative survival outcomes using the following covariates: age, ASA, BMI, sex, previous bladder cancer, hydronephrosis, tumor location, diagnostic ureteroscopy performed, neoadjuvant chemotherapy, year of surgery, perioperative intravesical chemotherapy, lymphadenectomy, T stage, tumor grade, variant histology, multifocality, number of lymph nodes removed, lymph node involvement, positive surgical margins, lymphovascular invasion, concomitant CIS, adjuvant chemotherapy, adjuvant radiotherapy.
Huang et al. 2023 [27] Balanced for - TNM stage; - Neo-adjuvant chemotherapy; - Prior bladder cancer; - Tumor location; - Body mass index; - ASA.	RNU vs. LNU 20 (8–37) vs. 29 (15–42.5), *p* = 0.006 (Median, IQR)	RNU vs. LNU 5-year OS 84.7% vs. 75%, *p* = 0.534; 5-year CSS 90.3% vs. 87.7%, *p* = 0.923; 5-year IV-RFS 62.9% vs. 65.2%, *p* = 0.363.

ND = No data.

**Figure 2:**
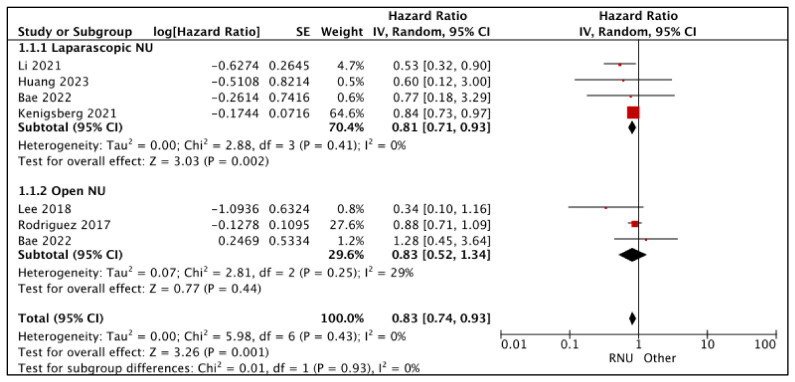
Primary outcomes: time-to-event analysis—overall survival [16,17,20,21,25,27].

- RNU vs. ONU

A total of three studies had comparative data considered suitable for a pooled analysis [16,17,25]. RNU and ONU had similar OS (HR: 0.83 (95% CI: 0.52, 1.34), I^2^ = 29%, *p*-0.44 (very low certainty)).

#### 3.1.2. Cancer-Specific Survival (CSS) (Figure 3)

- RNU vs. LNU

A total of two studies had comparative data considered suitable for a pooled analysis [21,25]. RNU and LNU had similar CSS (HR: 0.76 (95% CI: 0.45, 1.29), I^2^ = 0%, *p*-0.31 (very low certainty)).

**Figure 3:**
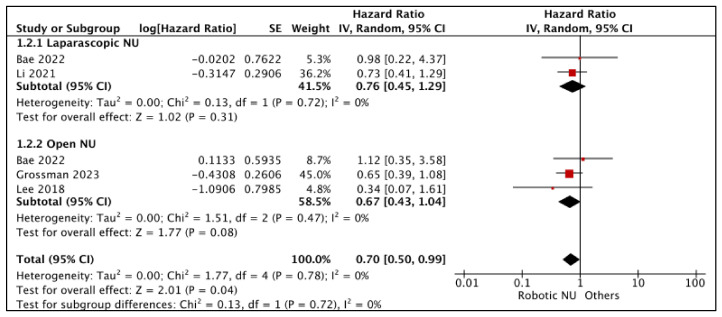
Primary outcomes: time-to-event analysis—cancer-specific survival [17,21,25,26].

- RNU vs. ONU

A total of three studies had comparative data considered suitable for a pooled analysis [17,25,26]. RNU and ONU had similar CSS (HR: 0.67 (95% CI: 0.43, 1.04), I^2^ = 0%, *p*-0.008 (very low certainty)).

#### 3.1.3. Disease-Free Survival (DFS) (Figure 4)

- RNU vs. LNU

Bae et al. compared DFS between RNU and LNU [25]. The study reported similar DFS (HR: 1.45 (95% CI: 0.68, 3.09), *p*-0.34 (very low certainty)).

**Figure 4:**
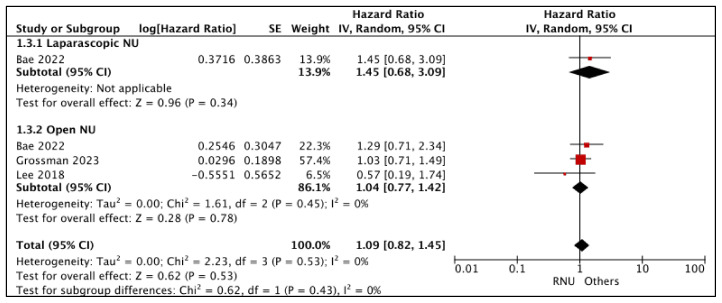
Primary outcomes: time-to-event analysis—disease-free survival [17,25,26].

- RNU vs. ONU

A total of three studies had comparative data considered suitable for a pooled analysis [17,25,26]. RNU and ONU had similar DFS (HR: 1.04 (95% CI: 0.77, 1.42), I^2^ = 0%, *p*-0.78 (very low certainty))

#### 3.1.4. Intravesical Recurrence-Free Survival (IV-RFS) (Figure 5)

- RNU vs. LNU

Li et al. reported similar IV-RFS between RNU and LNU (HR: 1.08 (95% CI: 0.75, 1.56), *p*-0.67 (very low certainty) [21].

**Figure 5:**
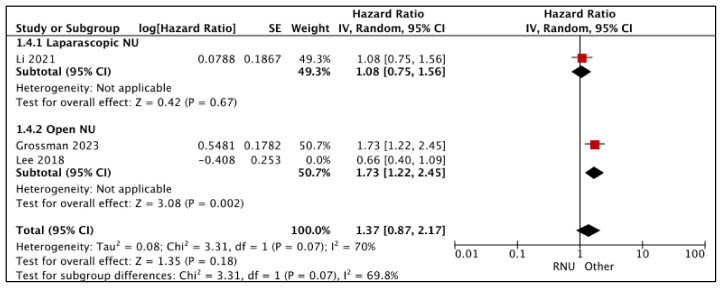
Primary outcomes: time-to-event analysis—intravesical recurrence-free survival [17,21,26].

- RNU vs. ONU

Grossman et al. reported an independent association between RNU as a worse predictor of IV-RFS when compared to ONU (HR-1.73 (95% CI: 1.22, 2.45) [26]. Other variables included in the Cox regression multivariate analysis were diagnostic URS, previous bladder cancer history, pre-operative bladder chemotherapy, and tumor location. Lee et al. [17] reported similar IV-RFS between RNU and ONU; however, appropriate confounders were not accounted for in the Cox regression multivariate analysis.

### 3.2. Secondary Outcomes

#### 3.2.1. Lymph Node Dissection Rates (LND) (Figure 6)

- RNU vs. LNU

A total of eight studies had data comparing LND rates between RNU and LNU [13,15,18,20,21,24,26,27], and six studies had a higher proportion of patients who underwent an LND in the RNU compared to the LNU cohort [13,18,20,21,24,27]. Melquist et al.performed LNDs in all patients [15]. Huang et al. found similar LND rates between the two cohorts [27].

**Figure 6:**
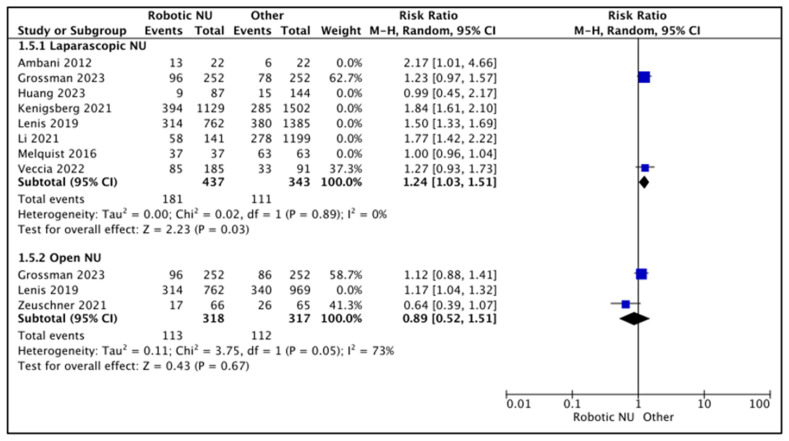
Secondary outcomes—lymphadenectomy rates [13,15,18,20,21,23,24,26,27].

A total of two studies were regarded as balanced and judged with a low risk of selection bias [24,26]. A pooled analysis of these studies suggested higher rates of LND in the RNU cohort (RR 1.24 (95% CI: 1.03, 1.51), I^2^ = 0%, *p*-0.03 (low certainty)).

- RNU vs. ONU

A total of three studies had data comparing LND rates between RNU and ONU [18,23,26], and two studies reported higher LND rates in the RNU cohort when compared to the ONU cohort [18,26]. Zeuschner et al. reported higher LND rates in the ONU cohort (25.8% RNU vs. 40% ONU) [23].

A total of two studies were regarded as balanced and judged with a low risk of selection bias [23,26]. A pooled analysis of these studies suggested variable rates of LND between the two cohorts (RR 0.89 (95% CI: 0.52, 1.51), I^2^ = 73%, *p*-0.67 (very low certainty)).

#### 3.2.2. Mean Lymph Node (LN) Count: (Figure 7)

A total of five studies had comparative data on the mean LN counts [17,20,23,24,26]. The mean LN counts in the RNU cohort ranged between 4.7 and 8.25. The mean LN counts in the LNU cohort ranged between 3.7 and 86.8. The mean LN counts in the ONU cohort ranged between 2.5 and 7.25.

**Figure 7:**
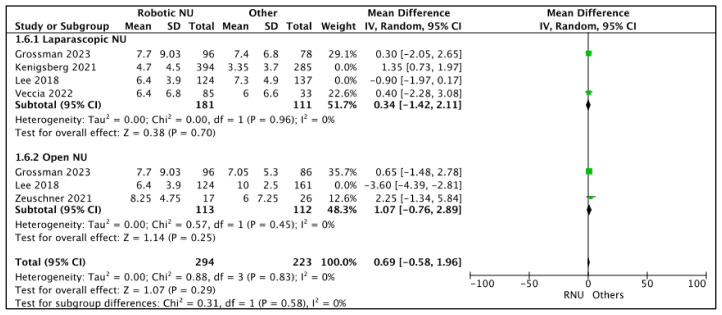
Secondary outcomes—mean lymph node counts [17,20,23,24,26].

- RNU vs. LNU

A total of four studies had data comparing the mean LN counts between RNU and LNU [17,20,24,26]. All studies had similar mean LN counts between the two cohorts, and two studies were regarded as balanced and judged with a low risk of selection bias [24,26]. A pooled analysis of these studies suggested similar mean LN counts between the two cohorts (MD 0.34 (95% CI: −1.42, 2.11), I^2^ = 0%, *p*-0.7 (low certainty)).

- RNU vs. ONU

A total of three studies had data comparing the mean LN counts between RNU and ONU cohorts [17,23,26], and two studies had similar mean LN counts between the two cohorts [23,26]. Lee et al. [17] reported a higher LN count in the RNU cohort. A total of two studies were regarded as balanced and judged with a low risk of selection bias [23,26]. A pooled analysis of these studies suggested similar mean LN counts between the two cohorts (MD 1.07 (95% CI: −0.76, 2.89), I^2^ = 0%, *p*-0.25 (low certainty)).

#### 3.2.3. Positive Margin (PSM) Rates (Figure 8)

- RNU vs. LNU

A total of nine studies had data comparing the PSM rates between RNU and LNU [13,15,18,19,20,24,25,26,27], and two studies were regarded as balanced and judged with a low risk of selection bias [24,26]. A pooled analysis of these studies suggested variable positive margin rates between the two cohorts (RR 0.89 (95% CI: 0.38, 2.09), I^2^ = 1%, *p*-0.79 (low certainty)).

**Figure 8:**
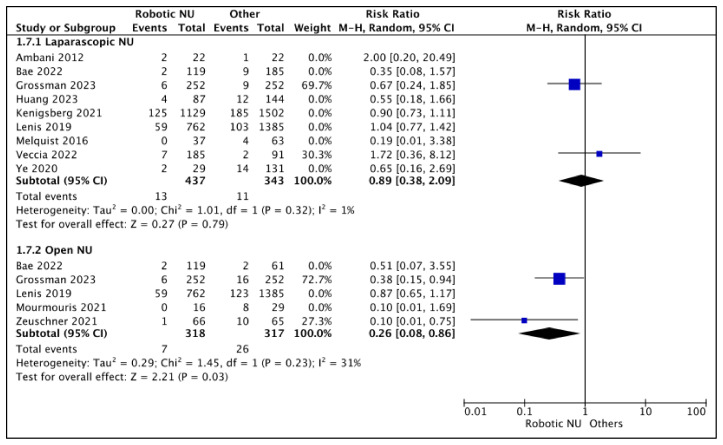
Secondary outcomes—positive margin rates [13,15,18,19,20,22,23,24,25,26,27].

- RNU vs. ONU

A total of five studies had comparative data [18,22,23,25,26], and two studies were considered balanced and judged with a low risk of selection bias [23,26]. A pooled analysis of these studies suggested lower positive margin rates in the RNU cohort (RR 0.29 (95% CI: 0.08, 0.86), I^2^ = 31%, *p*-0.03 (low certainty)).

#### 3.2.4. Post-NU Intravesical Chemotherapy Rates (Figure 9)

- RNU vs. LNU

A total of three studies had data comparing post-NU intravesical chemotherapy rates between RNU and LNU [19,24,26], and two studies were regarded as balanced and judged with a low risk of selection bias [24,26]. A pooled analysis of these studies suggested variable post-NU intravesical chemotherapy rates between the two cohorts (RR 0.98 (95% CI: 0.59, 1.62), I^2^ = 61%, *p*-0.79 (very low certainty)).

**Figure 9:**
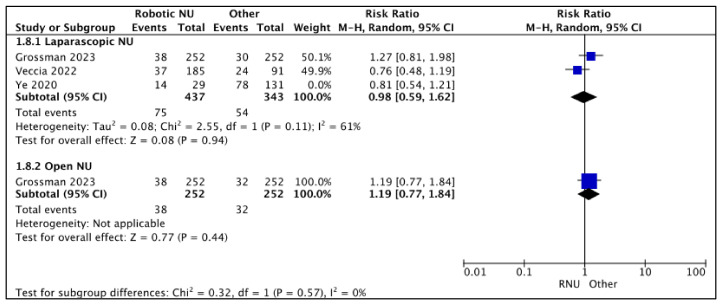
Secondary outcomes—post-NU intravesical chemotherapy rates [19,24,26].

- RNU vs. ONU

Only one study reported similar post-NU intravesical chemotherapy rates between RNU and ONU RR 1.19 (95% CI: 0.77, 1.84), *p*-0.44 (low certainty) [26].

### 3.3. Quality of Evidence and Certainty of Outcomes (Figure 10, Table 5)

A total of three studies were judged as having a low risk of selection bias, as a propensity-based matched pair analysis was performed [23,24,26]. All remaining studies were judged as having a high risk of bias. Additionally, 14 studies were judged as having a high risk of bias for performance and detection bias [13,14,15,16,17,18,19,20,21,23,24,25,26,27]. Only one was judged as having an unclear risk of bias, as the study was reported as a prospective study; however, no information was provided on blinding [22]. All studies were judged as having a high risk of bias for selective reporting, as no protocol was present.

**Figure 10:**
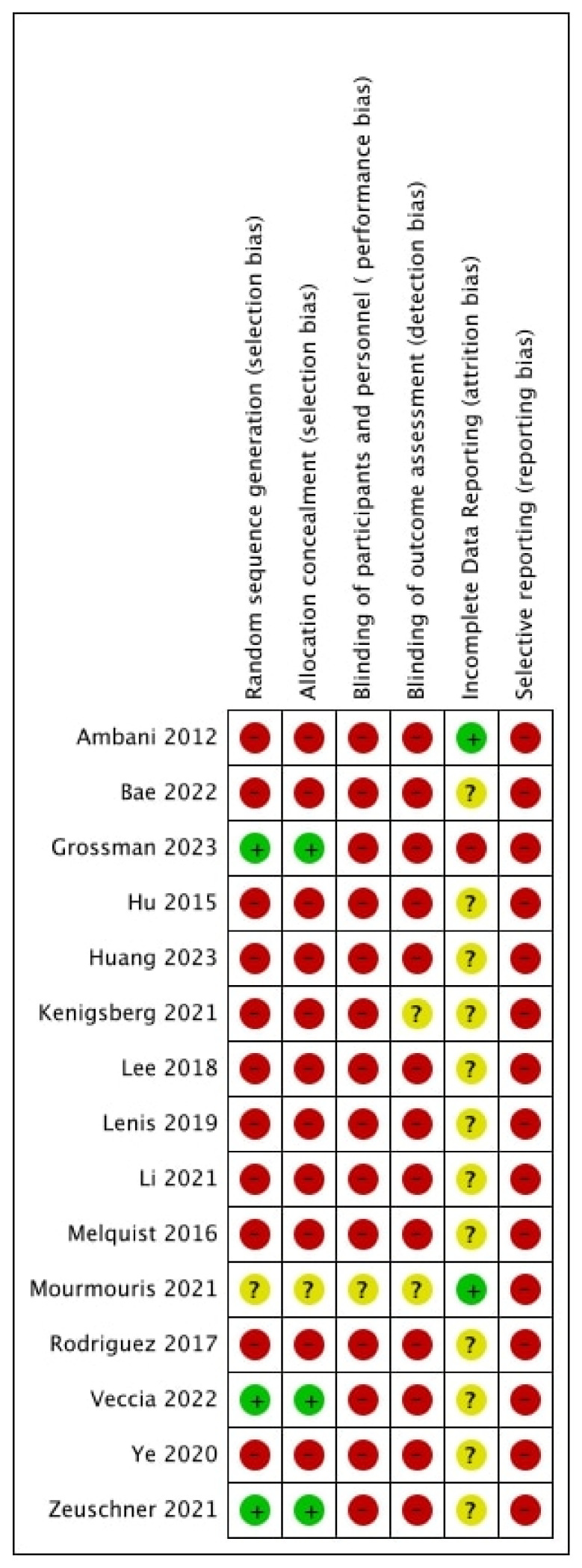
Risk of bias [13,14,15,16,17,18,19,20,21,22,23,24,25,26,27].

**Table 5:** GRADE assessment.

Primary and Secondary Outcomes Comparing RNU to ONU/LNU for Suspected UTUC					
Patient or Population: Patients with Suspected Non-Metastatic UTUC Setting: Hospital Intervention: RNU Comparison: LNU/ONU					
Outcomes	№ of Participants (Studies) Follow-Up	Certainty of the Evidence (GRADE)	Relative Effect (95% CI)	Anticipated Absolute Effects	
				Risk with Placebo	Risk Difference with Primary Outcomes
Time-to-Event Analysis: Overall Survival—Laparoscopic NU	(4 observational studies)	⨁◯◯◯ Very low ^a,b^	HR 0.81 (0.71 to 0.93)	0 per 1000	-- per 1000 (-- to --)
Time-to-Event Analysis: Overall Survival—Open NU	(3 observational studies)	⨁◯◯◯ Very low ^a,b^	HR 0.83 (0.52 to 1.34)	0 per 1000	-- per 1000 (-- to --)
Time-to-Event Analysis: Cancer-Specific Survival—Laparoscopic NU	(2 observational studies)	⨁◯◯◯ Very low ^a,b^	HR 0.76 (0.45 to 1.29)	0 per 1000	-- per 1000 (-- to --)
Time-to-Event Analysis: Cancer-Specific Survival—Open NU	285 (3 observational studies)	⨁◯◯◯ Very low ^a,b^	HR 0.67 (0.43 to 1.04)	0 per 1000	-- per 1000 (-- to --)
Time-to-Event Analysis: Disease-Free Survival—Laparoscopic NU	(1 observational study)	⨁◯◯◯ Very low ^a,b,c^	HR 1.45 (0.68 to 3.09)	0 per 1000	-- per 1000 (-- to --)
Time-to-Event Analysis: Disease-Free Analysis—Open NU	(3 observational studies)	⨁◯◯◯ Very low ^a,b^	HR 1.04 (0.77 to 1.42)	0 per 1000	-- per 1000 (-- to --)
Time-to-Event Analysis: Intravesical Recurrence-Free Survival—Laparoscopic NU	(1 observational study)	⨁◯◯◯ Very low ^a,b^	HR 1.08 (0.75 to 1.56)	0 per 1000	-- per 1000 (-- to --)
Time-to-Event Analysis: Intravesical Recurrence-Free Survival—Open NU	(1 observational study)	⨁◯◯◯ Very low ^a,b^	HR 1.73 (1.22 to 2.45)	0 per 1000	-- per 1000 (-- to --)
Lymphadenectomy Rates—Laparoscopic NU	780 (2 observational studies)	⨁⨁◯◯ Low ^a,b^	RR 1.24 (1.03 to 1.51)	324 per 1000	78 more per 1000 (10 more to 165 more)
Lymphadenectomy Rates—Open NU	635 (2 observational studies)	⨁◯◯◯ Very low ^a,b,d^	RR 0.89 (0.52 to 1.51)	353 per 1000	39 fewer per 1000 (170 fewer to 180 more)
Median Lymph Node Count—Laparoscopic NU	292 (2 observational studies)	⨁⨁◯◯ Low ^a,b^	-	The mean median lymph node count—laparoscopic NU was 0	MD 0.34 higher (1.42 lower to 2.11 higher)
Median Lymph Node Count—Open NU	225 (2 observational studies)	⨁⨁◯◯ Low ^a,b^	-	The mean median lymph node count—open NU was 0	MD 1.07 higher (0.76 lower to 2.89 higher)
Positive Margin Rates—Laparoscopic NU	780 (2 observational studies)	⨁⨁◯◯ Low ^a,b^	RR 0.89 (0.38 to 2.09)	32 per 1000	4 fewer per 1000 (20 fewer to 35 more)
Positive Margin Rates—Open NU	635 (2 observational studies)	⨁⨁◯◯ Low ^a,b^	RR 0.26 (0.08 to 0.86)	82 per 1000	61 fewer per 1000 (75 fewer to 11 fewer)
Proportion of Patients Receiving Intra-vesical Chemotherapy—Laparoscopic NU	780 (2 observational studies)	⨁◯◯◯ Very low ^a,b,c^	RR 0.98 (0.59 to 1.62)	157 per 1000	3 fewer per 1000 (65 fewer to 98 more)
Proportion of Patients Receiving Intra-vesical Chemotherapy—Open NU	504 (1 observational study)	⨁⨁◯◯ Low ^a,b^	RR 1.19 (0.77 to 1.84)	127 per 1000	24 more per 1000 (29 fewer to 107 more)
The risk in the intervention group (and its 95% confidence interval) is based on the assumed risk in the comparison group and the relative effect of the intervention (and its 95% CI). CI: Confidence interval; HR: hazard ratio; MD: mean difference; RR: risk ratio.					
GRADE Working Group grades of evidence High certainty: We are very confident that the true effect lies close to that of the estimate of the effect. Moderate certainty: We are moderately confident in the effect estimate; the true effect is likely to be close to the estimate of the effect, but there is a possibility that it is substantially different. Low certainty: Our confidence in the effect estimate is limited; the true effect may be substantially different from the estimate of the effect. Very low certainty: We have very little confidence in the effect estimate; the true effect is likely to be substantially different from the estimate of effect.					

^a^ Retrospective design, ^b^ no protocol, ^c^ wide CI, ^d^ statistical and clinical heterogeneity.

The certainty of the evidence for all pooled outcome measures was low or very low. The reasons for downgrading were study limitations, heterogeneity, and imprecision.

## 4. Discussion

The outcomes of this review suggest that RNU offers oncological efficacy comparable with that of ONU, except for IV-RFS. Furthermore, RNU appears to outperform LNU for certain oncological parameters, such as OS and the proportion of patients who receive lymph node dissections. However, the surgical approach did not seem to influence the number of lymph nodes retrieved.

Patient-specific factors, such as age, coexisting co-morbidities, and performance status, are important surgical considerations in patients with UTUC. A substantial proportion of them are in their seventh decade, potentially accompanied by frailty [1]. Elderly patients with comorbidities may not be suitable candidates for this approach. An estimated 60% of patients with UTUC will also have invasive disease at presentation and will require a multimodal treatment strategy with systemic anticancer therapy [1]. A phase 3, open-label, randomized controlled trial reported that adjuvant gemcitabine–platinum combination chemotherapy within 90 days following an NU improves an individual’s disease-free survival [28]. The potential for neoadjuvant chemotherapy is also currently being explored in high-risk disease [29]. It is, therefore, imperative that the physiological impact of major surgical intervention, particularly in a relatively co-morbid cohort of patients, does not hinder an individual’s ability to receive systemic chemotherapy. Furthermore, delayed recovery could hinder the administration of systemic anticancer therapy within a 90-day threshold. This review’s findings suggest that RNU’s OS, CSS, and DFS are on par with ONU, irrespective of the disease stage. RNU, therefore, retains the advantages of minimally invasive surgery without compromising the oncological efficacy and, therefore, is likely to be offered to a wider cohort of patients with UTUC. Furthermore, well-balanced studies in this review have reported superior PSM rates with RNU when compared to ONU.

The role of lymph node dissection (LND) during NU remains a topic of uncertainty, with conflicting evidence. Dominguez-Escrig et al. [30], in a systematic review of six studies, suggested that 14.3% to 40% of patients may have nodal involvement following LND in clinically node-negative cases. The review further reported that template LND might enhance cancer-specific survival (CSS) in cases of T2 or higher disease in the renal pelvis. The plausible benefits of LND should again be balanced against the increased morbidity associated with it. Minimally invasive approaches can mitigate some of the morbidity associated with LND. However, the widespread adoption of LND with conventional laparoscopic approaches may be limited due to technical challenges. The technical advantages of a robotic approach are likely to allow for a broader cohort of surgeons to confidently undertake LNDs in these patients. This hypothesis is reflected in the outcomes of this review, demonstrating that a higher proportion of patients underwent an RNU during an NU compared to an LNU.

Notably, one study within this review indicated inferior bladder cancer-free recurrence rates (BCFRs) in RNU compared to ONU [26]. Similar observations have been reported in laparoscopic techniques. However, it was hypothesized that these findings could be attributed to suboptimal techniques used in addressing the lower end of the bladder. The robotic approach offers the advantage of robust bladder cuff excision (BCE) with excellent bladder closure and, therefore, the authors are unable to provide a rationale behind these observations, warranting further investigation. A lack of detail regarding lower-end techniques and early experience could be contributing factors. Furthermore, issues such as immortal time bias while assessing metachronous bladder cancer recurrence in patients with locally advanced UTUC remain an unaddressed issue in the literature and may be a plausible explanation for these observations [31].

This review has a few limitations. This review highlights a paucity of high-quality data on the subject, a conclusion consistent with a few systematic reviews over the past decade [2,5]. Almost all studies in this review adopted a retrospective design and, consequently, most domains in the ROB assessment were judged as having a high or unclear risk of bias. To mitigate some of these biases, the authors presented a pooled analysis only for the primary studies where a matched pair analysis or a multivariate Cox regression analysis for time-to-event outcomes with appropriate covariates was performed. The authors would like to emphasize that this approach was not explicitly stated in our protocol and, therefore, should be regarded as a deviation from the protocol. Despite this approach, the authors recommend that a degree of caution remains imperative while interpreting these results, which is reflected in the very low certainty of outcomes in the GRADE evaluation. Additionally, the authors have not presented the sub-analysis and additional outcome data, as originally stated in the protocol. The authors intend to encompass these data as part of an upcoming systematic review document.

Generating high-quality level-1 evidence assessing surgical approaches for UTUC is admittedly complex. The authors would, however, like to emphasize that such evidence has been achieved in intricate surgical domains, such as radical cystectomy for bladder cancers [32]. The survival outcomes for UTUC continue to be unfavorable and, therefore, it is incumbent on the research bodies to prioritize and invest in UTUC research to address existing knowledge gaps and refine surgical strategies for these patients.

## 5. Conclusions

RNU offers oncological efficacy comparable with that of ONU, except for intravesical recurrence-free survival (IV-RFS). RNU has fewer positive surgical margin rates compared to ONU in well-balanced studies. RNU appears to outperform LNU for certain oncological parameters, such as OS and the proportion of patients who receive lymph node dissections. The quality of evidence comparing surgical techniques for UTUC has remained poor in the last decade. It is incumbent on the research bodies to prioritize and invest in UTUC research. Focused effort in addressing the existing knowledge gaps and refining surgical strategies for UTUC is vital.

## Figures and Tables

**Table 1:** Design of included studies.

Author	Type of Study	Groups Compared	Centers	Study Characteristics	Site of Study
Ambani et al. 2012 [13]	Retrospective	Robotic vs. lap (including hand-assisted)	Single	Matched pair (tumor stage and age)	USA
Hu et al. 2015 [14]	Retrospective	Robotic vs. hand-assisted lap	Single	Matched pair (tumor location, sex, and age)	Taiwan
Melquist et al. 2016 [15]	Retrospective	Robotic vs. lap + open lower end	Single	Consecutive patients	USA
Rodriguez et al. 2017 [16]	Retrospective	Robotic vs. lap vs. open	Population database (NCDB)	-	USA
Lee et al. 2018 [17]	Retrospective	Robotic vs. lap vs. open	Population database (NCDB)	-	USA
Lenis et al. 2019 [18]	Retrospective	Robotic vs. lap vs. open	Single	-	Seoul, Republic of Korea
Ye et al. 2020 [19]	Retrospective	Robotic vs. lap	Single	-	China
Kenigsberg et al. 2021 [20]	Retrospective	Robotic vs. lap	Population database (NCDB)	-	USA
Li et al. 2021 [21]	Retrospective	Robotic vs. lap vs. hand-assisted lap	Multicenter (15 centers)	-	Taiwan
Mourmouris et al. 2021 [22]	Prospective	Robotic vs. open	Two centers	Consecutive patients	Greece, Turkey
Zeuschner et al. 2021 [23]	Retrospective	Robot-assisted vs. open	Single	Propensity-score matched pair (1:1)	Germany
Veccia et al. 2022 [24]	Retrospective	Robotic vs. lap	Multicenter (17 centers)	Propensity-score matched pair (2:1)	Worldwide
Bae et al. 2022 [25]	Retrospective	Robotic vs. lap vs. open	Single	-	Republic of Korea
Grossmann et al. 2023 [26]	Retrospective	Robotic vs. lap vs. open	Multicenter (21 centers)	1:1:1 Propensity-score matching (PSM) analysis	Europe, Asia, USA
Huang et al. 2023 [27]	Retrospective	Robotic vs. lap	Single	-	Taiwan

**Table 2:** Demographics of included studies.

Author	Groups	Age	*p*-Value	Gender—Males (n, %)	*p*-Value	Hydronephrosis	*p*-Value	BMI	*p*-Value
Ambani et al. 2012 [13]	RNU vs. LNU	70.1 ± 2.2 vs. 70.8 ± 2.2 (Mean, SD)	0.53	14 (64%) vs. 16 (73%)	0.71	ND		ND	
Hu et al. 2015 [14]	RNU vs. LNU	70.4 ± 6.3 vs. 69.6 ± 5.7 (Mean, SD)	0.646	5 (27.8%) vs. 5 (27.8%)	1	12 (66.7%) vs. 11 (61.1%)	1	23.8 ± 3.4 vs. 25.0 ± 4.9 (Mean, SD)	0.411
Melquist et al. 2016 [15]	RNU vs. LNU	68 (63.6–73.6) vs. 72.6 (65.8–81.8) (Median, IQR)	0.06	26 (70%) vs. 36 (57%)	0.2	ND		28 (26.1–32.6) vs. 28 (25.1–31.3) (Median, IQR)	0.34
Rodriguez et al. 2017 [16]	RNU vs. LNU vs. ONU	70.3 vs. 71.1 vs. 71 (Mean)	0.01	62.40% vs. 59.10% vs. 59.10%	0.03	ND		ND	
Lee et al. 2018 [17]	RNU vs. LNU vs. ONU	67.6 ± 11.3 vs. 68.6 ± 10.4 vs. 67.5 ± 10.2 (Mean, SD)	0.642	85 (68.5%) vs. 97 (70.8%) vs. 117 (72.7%)	0.75	83 (68.0%) vs. 90 (65.7%) vs. 115 (71.9%)	0.577	24.6 ± 2.9 vs. 23.9 ± 3.6 vs. 23.7 ± 2.8 (Mean, SD)	0.062
Lenis et al. 2019 [18]	RNU vs. LNU vs. ONU	70.0 ± 10.9 vs. 70.6 ± 10.4 vs. 70.6 ± 10.5 (Mean, SD)	0.48	469 (61.6%) vs. 772 (55.7%) vs. 537 (55.4%)	0.02	ND		ND	
Ye et al. 2020 [19]	RNU vs. LNU	71 (48–84) vs. 66 (44–83) (Median, IQR)	0.924	21 (72.4%) vs. 82 (62.6%)	0.394	ND		ND	
Kenigsberg et al. 2021 [20]	RNU vs. LNU	71.4 vs. 72.7 (Mean)	<0.001	741 (65.6%) vs. 909 (60.5%)	0.007	ND		ND	
Li et al. 2021 [21]	RNU vs. LNU	PD	0.140	61 (43.3%) vs. 194 (42.4%)	0.916	61 (43.3%) vs. 250 (54.6%)	<0.001	ND	
Mourmouris et al. 2021 [22]	RNU vs. ONU	68.12 ± 9.0 vs. 67.12 ± 12.19 (Mean, SD)	0.8	14 (87.5%) vs. 24 (82.8%)	1	ND		25.2 ± 1.85 vs. 26.54 ± 1.95 (Mean, SD)	0.12
Zeuschner et al. 2021 [23]	RNU vs. ONU	70.5 (39–86) vs. 74 (51–92) (Median, range)	0.147	35 (53%) vs. 43 (66.2%)	0.126	3 (4.5%) vs. 10 (15.4%)	0.062	26 (18–35) vs. 26 (17–40) (Median, range)	0.987
Veccia et al. 2022 [24]	RNU vs. LNU	72 (65–78) vs. 71 (64–77) (Median, IQR)	0.44	106 (57.8%) vs. 55 (60.4%)	0.69	72 (38.9%) vs. 44 (48.3%)	0.33	26.3 (24.2–28.7) vs. 26.6 (24.7–28.5) (Median, IQR)	0.57
Bae et al. 2022 [25]	RNU vs. LNU vs. ONU	68.5 ± 9.1 vs. 67.6 ± 9.6 vs. 69.7 ± 9.4 (Mean, SD)	0.295	85 (71.4%) vs. 131 (70.8%) vs. 41 (67.2%)	0.830	ND		25.2 ± 3.8 vs. 24.6 ± 3.2 vs. 25.3 ± 3.2 (Mean, SD)	0.171
Grossmann et al. 2023 [26]	RNU vs. LNU vs. ONU	70 (62–77) vs. 72 (65–78) vs. 73 (66–78) (Median, IQR)	0.05	168 (66.7%) vs. 170 (67.4%) vs. 167 (66.3%)	>0.9	114 (45%) vs. 111 (44%) vs. 91 (36%)		26 (23.4–29) vs. 26 (23–29) vs. 25.6 (22.3–28.9) (Median, IQR)	0.3
Huang et al. 2023 [27]	RNU vs. LNU	72 (63–80) vs. 72 (63–81) (Median, IQR)	0.987	47 (54%) vs. 76 (52.8%)	0.892	ND		23.9 (21.6–25.9) vs. 24 (21.1–26.9) Median, IQR	0.703

ND = No data, PD = parameters different.

**Table 3:** Tumor characteristics of included studies.

Author	Groups	TNM Stage			Tumor Grade			Tumor Location			
		<T2	≥T2	*p*-Value	Low	High	*p*-Value	Kidney	Ureter	Both	*p*-Value
Ambani et al. 2012 [13]	RNU vs. LNU	55% vs. 55%	45% vs. 45%	ND	ND	ND	ND	64% vs. 64%	27% vs. 27%	9% vs. 9%	0.32
Hu et al. 2015 [14]	RNU vs. LNU	44.5% vs. 50%	55.5% vs. 50%	0.165	5.56% vs. 16.7%	94.4% vs. 83.3%	0.603	55.6% vs. 55.6%	27.8% vs. 27.8%)	16.7% vs. 16.7%	1
Melquist et al. 2016 [15]	RNU vs. LNU	75% vs. 56%	25% vs. 45%	0.43	ND	ND	ND	57% vs. 46%	24% vs. 37%	19% vs. 17%	0.44
Rodriguez et al. 2017 [16]	RNU vs. LNU vs. ONU	55.4% vs. 54.3% vs. 52.7%	44.5% vs. 45.7% vs. 47.3%	0.02	ND	69% vs. 69.8% vs. 72.9%	0.01	ND	32% vs. 30.6% vs. 34.4%	ND	<0.01
Lee et al. 2018 [17]	RNU vs. LNU vs. ONU	33.9% vs. 35.8% vs. 23%	66.1% vs. 64.3% vs. 77%	0.001	PD	PD	0.177	50.8% vs. 56.9% vs. 52.2%	42.7% vs. 38.7% vs. 33.5%	6.5% vs. 4.4% vs. 14.3%	0.013
Lenis et al. 2019 [18]	RNU vs. LNU vs. ONU	51.4% vs. 47.9% vs. 43.5%	46.7% vs. 50.1% vs. 54.1%	<0.01	29.7% vs. 25.6% vs. 23.4%	70.3% vs. 74.4% vs. 76.6%	0.01	71.9% vs. 70.7% vs. 65.9%	28.1% vs. 29.3% vs. 34.1%	ND	0.01
Ye et al. 2020 [19]	RNU vs. LNU	41.3% vs. 37.4%	58.7% vs. 62.6%	0.842	44.8% vs. 32.1%	55.2% vs. 67.9%	0.201	37.9% vs. 44.3%	51.7% vs. 45.8%	10.4% vs. 9.9%	0.817
Kenigsberg et al. 2021 [20]	RNU vs. LNU	62% vs. 60.3%	38.1% vs. 39.7%	0.456	PD	PD	0.456	ND	ND	ND	ND
Li et al. 2021 [21]	RNU vs. LNU	45.3% vs. 44.5%	54.6% vs. 55.5%	0.906	12.8% vs. 13.8%	87.2% vs. 86.2%	0.015	59.3% vs. 48.7%	23.6% vs. 38.4%	17.1% vs. 12.9%	<0.001
Mourmouris et al. 2021 [22]	RNU vs. ONU	50% vs. 48.3%	50% vs. 51.7%	0.01	56.3% vs. 27.6%	43.7% vs. 72.4%	0.06	62.5% vs. 48.3%	37.5% vs. 51.7%	ND	0.06
Zeuschner et al. 2021 [23]	RNU vs. ONU	34.8% vs. 30.8%	65.1% vs. 69.2%	NS	ND	ND	ND	DNC	DNC	DNC	DNC
Veccia et al. 2022 [24]	RNU vs. LNU	45.4% vs. 42.8%	54.3% vs. 57.2%	0.11	ND	55.3% vs. 70.4%	0.23	69% vs. 64.6%	ND	ND	
Bae et al. 2022 [25]	RNU vs. LNU vs. ONU	42% vs. 49.3% vs. 47.5%	57.9% vs. 50.8% vs. 52.5%	0.742	PD	PD	0.222	44.5% vs. 42.2% vs. 41%	45.4% vs. 49.7% vs. 52.5%	10.1% vs. 8.1% vs. 6.6%	0.863
Grossmann et al. 2023 [26]	RNU vs. LNU vs. ONU	53% vs. 57% vs. 53%	47% vs. 43% vs. 46%	0.8	26% vs. 25% vs. 23%	74% vs. 75% vs. 77%	0.6	65% vs. 65% vs. 63%	33% vs. 32% vs. 32%	2% vs. 3.2% vs. 4.8%	0.5
Huang et al. 2023 [27]	RNU vs. LNU	47.1% vs. 53.5%	52.8% vs. 45.8%	0.593	5.7% vs. 8.3%	94.3% vs. 91%	0.606	52.9% vs. 52.1%	31% vs. 38.2%	16.1% vs. 9.7%	0.262

ND = No data, PD = parameters different, DNC = data not clear, NS = not significant.

## Supplementary Materials

The following supporting information can be downloaded at: https://www.mdpi.com/article/10.3390/cancers15204926/s1, Supplementary File S1—Search strategy.
