# Supplementary material for: Oncological Efficacy of Robotic Nephroureterectomy vs. Open and Laparoscopic Nephroureterectomy for Suspected Non-Metastatic UTUC—A Systematic Review and Meta-Analysis
Source: Cancers (Basel). 2023 Oct 10;15(20):4926. doi: 10.3390/cancers15204926 (PMC10605607; doi:10.3390/cancers15204926)
Supplement: Supplementary file 1 [file cancers-15-04926-s001.zip › cancers-2617061-supplementary.pdf]

# Search strategies TCC

MEDLINE (OVID)

1. exp Carcinoma, Transitional Cell/
2. exp Ureteral Neoplasms/
3. ((upper tract or renal pelv$ or ureter$ or calice$) adj3 (urothelial or tcc or transitional or carcinoma$ or tumo?r$ or cancer$ or neoplas$)).tw.
4. or/1-3
5. exp Surgery/
6. exp nephrectomy/
7. exp partial nephrectomy/
8. nephroureterectom$.tw.
9. ((ureteral or percutaneous or surgical or ureteroscopic or endoscopic) adj3 (resection or management or fulguration)).tw.
10. (ONU or LNU).tw.
11. ((radical or open or laparoscop$) adj3 (surg$ or nephro$ or nephrec)).tw.
12. partial nephrectomy.tw.
13. exp Electrocoagulation/
14. exp Laser Therapy/
15. re-anastomosis.tw.
16. electrocaut$.tw.
17. ((segmental or bladder cuff) adj2 resection).mp.
18. or/5-17
19. 4 and 18
20. randomized controlled trial.pt.
21. controlled clinical trial.pt.
22. randomized.ab.
23. placebo.ab.
24. drug therapy.fs.
25. randomly.ab.
26. trial.ab.
27. groups.ab.
28. or/20-27
29. humans.sh.

30. 28 and 29
31. 19 and 30

# Embase (OVID)

1. Transitional Cell Carcinoma/
2. exp Ureter Tumor/
3. ((upper tract or renal pelv$ or ureter$ or calice$) adj3 (urothelial or tcc or transitional or carcinoma$ or tumo?r$ or cancer$ or neoplas$)).tw.
4. or/1-3
5. exp SURGERY/
6. exp nephrectomy/
7. exp partial nephrectomy/
8. nephroureterectom$.tw.
9. ((ureteral or percutaneous or surgical or ureteroscopic or endoscopic) adj3 (resection or management or fulguration)).tw.
10. (ONU or LNU).tw.
11. ((radical or open or laparoscop$) adj3 (surg$ or nephro$ or nephrec)).tw.
12. partial nephrectomy.tw.
13. exp ELECTROCOAGULATION/
14. exp Low Level Laser Therapy/
15. exp Cauterization/
16. (re-anastomosis or electrocaut$).tw.
17. ((segmental or bladder cuff) adj2 resection).tw.
18. or/5-17
19. 4 and 18
20. Crossover Procedure/
21. double-blind procedure/
22. randomized controlled trial/
23. single-blind procedure/
24. (random$ or factorial$ or crossover$ or cross over$ or placebo$ or assign$ or allocat$ or volunteer$).mp.
25. ((doubl$ or singl$) adj blind$).mp.
26. or/20-25
27. 19 and 26

## Cochrane Library (Wiley)

1. MeSH descriptor Carcinoma, Transitional Cell explode all trees
2. MeSH descriptor Ureteral Neoplasms explode all trees
3. (upper tract or renal pelv* or ureter* or calice*):kw,ti,ab NEAR/3 (urothelial or tcc or transitional or carcinoma* or tumor* or tumour* or cancer* or neoplasm*):kw,ti,ab
4. (#1 OR #2 OR #3)
5. MeSH descriptor Surgical Procedures, Operative explode all trees
6. MeSH descriptor Nephrectomy explode all trees
7. (nephrouereterectom* or nephrectom*):kw,ti,ab
8. (ureteral or percutaneous or surgical or ureteroscopic or endoscopic):kw,ti,ab NEAR/3 (resection or management or fulguration):kw,ti,ab
9. (ONU or LNU):kw,ti,ab
10. (radical or open or laparoscop*):kw,ti,ab NEAR/3 (surg* or nephro* or nephrec*):kw,ti,ab
11. MeSH descriptor Electrocoagulation explode all trees
12. MeSH descriptor Laser Therapy explode all trees
13. re-anastomosis:kw,ti,ab OR electrocaut*:kw,ti,ab
14. (segmental or bladder cuff):kw,ti,ab NEAR/2 (resection):kw,ti,ab
15. (#5 OR #6 OR #7 OR #8 OR #9 OR #10 OR #11 OR #12 OR #13 OR #14)

## Web of Science

1. TS= clinical trial* OR TS=research design OR TS=comparative stud* OR TS=evaluation stud* OR TS=controlled trial* OR TS=follow-up stud* OR TS=prospective stud* OR TS=random* OR TS=placebo* OR TS=(single blind*) OR TS=(double blind*)
2. TS=(transitional cell carcinoma)
3. TS=(upper tract urothelial or renal pelvis or ureter or ureteral) SAME TS=(cancer* or carcinoma* or tumor* or tumour* or neoplas*)
4. #3 OR #2
5. TS=(surgery)
6. TS=(nephroureterectomy or resection or fulguration or electro* or laser)
7. #6 OR #5
8. #7 AND #4 AND #1

# CINAHL (Ebsco)

1. (MH "Bladder Neoplasms")
2. bladder* N3 cancer*
3. bladder* N3 neoplasm*
4. ureter* N3 neoplasm*
5. ureter* N3 cancer*
6. (transitional cell ) or tcc
7. S1 or S2 or S3 or S4 or S5 or S6
8. (MH "Surgery, Operative+")
9. (MH "Nephrectomy")
10. nephroureterectom* or nephrectom*
11. partial or radical or open or laparoscop*
12. resect* or fulgarat*
13. S8 or S9 or S10 or S11 or S12
14. S7 and S13
15. ( (MH "Random Assignment") or (MH "Random Sample+") or (MH "Crossover Design") or (MH "Clinical Trials+") or (MH "Comparative Studies") or (MH "Control (Research)+") or (MH "Control Group") or (MH "Factorial Design") or (MH "Quasi-Experimental Studies+") or (MH "Placebos") or (MH "Meta Analysis") or (MH "Sample Size") or (MH "Research, Nursing") or (MH "Research Question") or (MH "Research Methodology+") or (MH "Evaluation Research+") or (MH "Concurrent Prospective Studies") or (MH "Prospective Studies") or (MH "Nursing Practice, Research-Based") or (MH "Solomon Four-Group Design") or (MH "One-Shot Case Study") or (MH "Pretest-Posttest Design+") or (MH "Static Group Comparison") or (MH "Study Design") or (MH "Clinical Research+") ) or ( clinical nursing research or random* or cross?over or placebo* or control* or factorial or sham* or meta?analy* or systematic review* or blind* or mask* or trial* )
16. S14 and S15

# British Nursing Index (OVID)

1. exp cancer/
2. exp "Urinary System and Disorders"/
3. ((bladder$ or upper tract or renal pelv$ or ureter$ or calice$) adj3 (urothelial or tcc or transitional or carcinoma$ or tumo?r$ or cancer$ or neoplas$)).tw.
4. 1 and 2
5. 3 or 4

6. exp surgery : operative/
7. (nephroureterectom$ or nephrectom$).tw.
8. ((ureteral or percutaneous or surgical or ureteroscopic or endoscopic) adj3 (resection or management or fulguration)).tw.
9. ((partial or radical or open or laparoscop$) adj3 (surg$ or nephro$ or nephrec)).tw.
10. 6 or 7 or 8 or 9
11. 5 and 10
12. Randomized controlled trial$.mp.
13. (clinic$ adj trial$1).tw.
14. ((singl$ or doubl$ or treb$ or tripl$) adj (blind$ or mask$)).tw.
15. (allocated adj2 random).tw.
16. placebo$.mp.
17. 12 or 13 or 14 or 15 or 16
18. 11 and 17

# LILACS

(Transitional or Transicionales or Transição or tcc) and (surgery or surgical or cirurg$ or cirugi$ or quirúrg$ or nephrectom$ or nefrectom$) and (Pt RANDOMIZED CONTROLLED TRIAL OR Pt CONTROLLED CLINICAL TRIAL OR Ab random$ OR Ab aleator$ OR Ab placebo$ OR Mh Clinical Trials as Topic OR Ti trial)

# Biomed Central

((random* OR trial* OR blind* OR placebo*)[tw]) AND ("transitional cell carcinoma"[TW] AND (surgery OR nephrectomy OR nephroureterectomy)[TW])

# BIOSIS

1. TS= clinical trial* OR TS=research design OR TS=comparative stud* OR TS=evaluation stud* OR TS=controlled trial* OR TS=follow-up stud* OR TS=prospective stud* OR TS=random* OR TS=placebo* OR TS=(single blind*) OR TS=(double blind*)
2. TS=(transitional cell carcinoma)
3. TS=(upper tract urothelial or renal pelvis or ureter or ureteral) SAME TS=(cancer* or carcinoma* or tumor* or tumour* or neoplas*)
4. #2 or #3
5. TS=(surgery)

6. TS=(nephroureterectomy or resection or fulguration or electro* or laser)
7. #5 or #6
8. #1 and #4 and #7

# Scopus

(TITLE-ABS-KEY("transitional cell carcinoma"))

OR (TITLE-ABS-KEY(urothelial OR tcc OR transitional) AND (carcinoma* OR tumo*r OR cancer* OR neoplas*))

AND (TITLE-ABS-KEY(surg* OR nephrectomy OR nephroureterectomy))

AND (TITLE-ABS-KEY(random* OR trial* OR blind* OR placebo*))

# AMED (OVID)

1. exp Bladder neoplasms/
2. ((bladder$ or upper tract or renal pelv$ or ureter$ or calice$) adj3 (urothelial or tcc or transitional or carcinoma$ or tumo?r$ or cancer$ or neoplas$)).tw.
3. 1 or 2
4. exp Surgery/
5. (nephroureterectom$ or nephrectom$).tw.
6. ((ureteral or percutaneous or surgical or ureteroscopic or endoscopic) adj3 (resection or management or fulguration)).tw.
7. ((partial or radical or open or laparoscop$) adj3 (surg$ or nephro$ or nephrec)).tw.
8. 4 or 5 or 6 or 7
9. 3 and 8
10. exp Randomized controlled trials/
11. (clinic$ adj trial$1).tw.
12. ((singl$ or doubl$ or treb$ or tripl$) adj (blind$ or mask$)).tw.
13. (allocated adj2 random).tw.
14. placebo$.mp.
15. 10 or 11 or 12 or 13 or 14
16. 9 and 15
